# The effect of early skin-to-skin contact after cesarean section on breastfeeding duration and development of atopic-allergic diseases

**DOI:** 10.18332/ejm/176213

**Published:** 2024-01-24

**Authors:** Yvonne Stephan, Hans-Helge Müller, Maritta Kühnert, Ivo Meinhold-Heerlein, Gentiana Ibrahimi, Maleen Reitz, Hannah Schemmann, Frank Oehmke, Siegmund Köhler, Harald Renz

**Affiliations:** 1 Institute of Laboratory Medicine and Pathobiochemistry Molecular Diagnostics, Philipps University Marburg, Marburg, Germany; 2 Faculty of Health, Technische Hochschule Mittelhessen, University of Applied Sciences, Giessen, Germany; 3 Department of Obstetrics and Gynecology, Division of Obstetrics, University Hospital Giessen and Marburg GmbH, Marburg, Germany; 4 Department of Obstetrics and Gynecology, University Hospital Giessen and Marburg GmbH, Giessen, Germany

**Keywords:** breastfeeding, cesarean section, skin contact, atopic-allergic diseases, skin-to-skin contact, atopic dermatitis

## Abstract

**INTRODUCTION:**

Breastfeeding to strengthen the immune system suggests allergy prevention as a possible option. The connection between breastfeeding and the development of atopic-allergic diseases is being discussed. The primary aim of this work was to investigate an association of the first early skin-to-skin contact following cesarean section with the development of atopic diseases within the 1st year of life.

**METHODS:**

The present study was conducted as a bicentric prospective cohort study in central Germany with a 15-month recruitment period. Data collection was by telephone interviews with a follow-up of 12 months. The statistical evaluation procedure was based on a hierarchical test of the association of early skin-to-skin contact between mother and child with the two main outcome measures. The primary outcome is the duration of breastfeeding. The second outcome is the onset of atopic-allergic disease within the 1st year of life.

**RESULTS:**

Mothers breastfed longer if they had skin-to-skin contact within the first 30 minutes postpartum [χ²(df=5) = 19.020, p=0.002], if they breastfed their newborns early immediately after birth (p<0.001), and if the first skin-to-skin contact lasted more than one hour [χ²(df=4) = 19.617, p<0.001]. Regarding atopic-allergic diseases, no significant effects of skin-to-skin contact were found in relation to disease development. Regarding breastfeeding, no significant effects of atopic-allergic diseases could be detected either.

**CONCLUSIONS:**

The results of this study reflect the benefits of skin-to-skin contact in the context of breastfeeding and atopic disease. The current scientific knowledge regarding skin contact and the development of atopic-allergic diseases should be extended and deepened.

## INTRODUCTION

Natural birth produces a cocktail of birth-promoting hormones in the body of the mother in labor. The hormonal situation after cesarean section compared to vaginal birth is different^1^. It is known that bonding has a beneficial effect on the breastfeeding relationship.

Recently, there has been a rise in allergies and atopic diseases among children^2-5^. There are considerable regional differences worldwide, and a significant increase in the prevalence of atopic dermatitis has been observed^2^. The primary cause is believed to be changes in environmental conditions and Western lifestyles^2,3^. It is believed that nutrition could potentially contribute to the development of atopic diseases since the microbiome in an infant’s gut is crucial for their ability to tolerate certain foods^6^.

Pathogens of atopic-allergic diseases are very complex and are currently being researched. A current issue in atopy research is directed at the microbiome formed by the environment on human skin and in the gut^3,7^. An altered microbiome has been found in children with atopic-allergic conditions. The microbiome is formed from various beneficial bacteria and is associated with a strong immune system. A diverse and healthy microbiome strengthens a healthy immune response to foreign antigens and microorganisms. Disruption of the microbiome leads to a defective immune response. Therefore, this relationship is the focus of further research^8^.

After a cesarean section, an altered microbiome has been found in the infants’ gut^9^. In addition, breastfeeding, for example, influences intestinal colonization in infants^8,10,11^. The support of breastfeeding and a longer duration of breastfeeding to support the immune system offer themselves as a possible allergy prevention, which can be a benefit for health later in life^5^.

Currently, several possibilities are being discussed to explain the link between breastfeeding and the development of atopic dermatitis^5,12^. Some studies have found a beneficial relationship between skin-to-skin contact and exclusive breastfeeding rates^13-15^. The possible beneficial effect of breastfeeding is known. Exclusive breastfeeding could reduce the prevalence of infectious diseases, food allergies, and diarrhea in the infant^16^. The study of Horta and Victora^17^ indicates that breastfeeding protects against diarrhea and respiratory infections. It is possible that breastfeeding, among other factors, has an impact on children’s long-term health^18^.

Because of the benefits of breastfeeding, the World Health Organization and professional societies suggest feeding infants exclusively with breast milk until they are at least six months old^2,19^. A study shows that the rate of exclusive breastfeeding decreases significantly earlier^20^. Consistently, the rate of exclusive breastfeeding decreases by 50% around the infant’s 4th month of life^21^. General data suggest that babies delivered by cesarean section have less skin-to-skin contact and are breastfed less in the hospital^22^. Early skin contact, as a factor that could have an influence on breastfeeding duration, has been the focus of research^14^.

Not only is a relationship between infant feeding and the occurrence of a disease from the atopic range discussed, but also a relationship with the mode of birth is suspected. One study found that infants born by cesarean section were more likely to require hospitalization for asthma than those infants born vaginally^23^. A meta-analysis of 8 studies with 36337 participants suggests no significant association between cesarean section and the development of atopic disease (OR=1.01; 95% CI: 0.95–1.08)^4^. A systematic review of 26 observational studies examined the possible association between the incidence of atopic disease and cesarean section. It was found that there was a 32 % increased risk of food allergy, a 23 % increased risk of allergic rhinitis, and an 18 % increased risk of asthma due to cesarean section, but no increased risk of atopic dermatitis (OR=1.03; 95% CI: 0.98–1.09)^12^.

Studies on the relationship between atopic-allergic disease and breastfeeding, as well as the mode of birth and disease in the infant, are controversial. The association between early skin-to-skin contact, especially after cesarean section, and the development of atopic disease has not been investigated. The present study focuses on the question of whether such a relationship exists. The primary objective of this work was to investigate a possible association of the first early skin-contact with the development of atopic allergic diseases within the first 12 months of life. A possible association between the first early skin-contact and the duration of breastfeeding up to 6 months of age is analyzed.

## METHODS

### Study design and study population

In the present study, skin-to-skin contact is considered as an exposure with a linking character between cesarean section and breastfeeding duration. For our study, early skin-to-skin contact means that the dry newborn is placed naked with the side of the abdomen on the mother’s bare chest or abdomen within 30 minutes of life. Early skin-to-skin contact after cesarean section is not specifically offered in the two hospitals where the study took place. The treatment depends on the clinic’s routine. Observations in the hospitals after cesarean section show that it is common for the mother to see her newborn briefly, and then take it directly to the pediatrician for initial care.

The present study was conducted as a bicentric prospective observational and longitudinal cohort study with a 15-month recruitment period at two university hospitals in Germany. Case number planning was performed with the help of statistical bioinformatic consulting. Two main outcome measures were established: duration of full breastfeeding and incidence of atopic dermatitis in the child within the first year of life. Two considerations were made that led to the identification of caseload in approximately 800 to 2000 mother–infant pairs. Especially with the inclusion of the two main target variables, a case number of around 1800 mother–child pairs would be desirable, and with 1.5 to 2 years of recruitment, this case number seemed achievable.

The study population consisted of mothers aged >18 years, at >37th + 0th gestational age, who delivered by elective cesarean section. Quantitative data were collected through telephone interviews using a standardized questionnaire. The development of the standardized interview guide was based on questions from previously developed questionnaires already used in studies^24-26^. To ensure the quality of the data collected in the questionnaire of the standardized interview guide, it was subjected to a pretest and adapted. The checklist immediately after birth was completed by the midwife in the surgery. The interviews at the following measurement points were conducted by the first author of this work with the assistance of a doctoral student in the Department of Medicine.

In cases where the women could no longer be reached by telephone, the women were contacted by e-mail. If there was no response, the women were contacted by postal mail. In some cases, this procedure was successful and further data could be collected.

**Figure 1:**
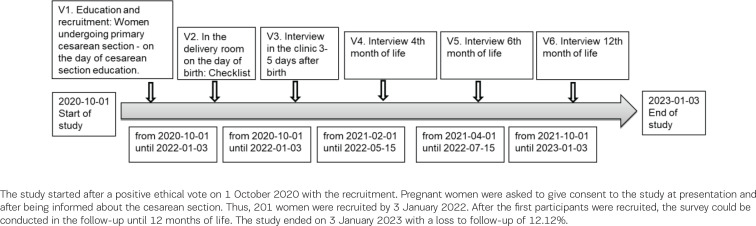
Association of the different components of this study

Data were collected at five measurement points with a follow-up of 12 months. The time points were immediately after birth, on the 3rd to 5th postpartum day of the clinical puerperium, and at the 4th, 6th, and 12th month of life. Figure 1 illustrates the study protocol. The first visit (V1) is the first contact with the study participant and includes personal education and recruitment to the study. The time was at least one day before the elective cesarean section, after the medical information for the planned cesarean section. The content of V1 refers to the information letter for parents, privacy information, and consent form for study participants. The second time point (V2) took place immediately after birth in the surgery and contains basic data on the mother and the newborn, information on birth and early skin-to-skin contact. The first questionnaire-guided telephone interview (V3) was conducted on the 3rd to 5th postpartum day before discharge. In the telephone interview guide for V3, baseline data, information about the birth, maternal health, atopy burden of the father and mother, breastfeeding preparation, first breastfeeding experience, breastfeeding behavior and support, infant feeding, sociodemographic questions, smoking during pregnancy and the mother’s feeding habits, were collected. The second telephone interview (V4) was conducted at the child’s 4th month of life. The contents of the V4 interview guide were breastfeeding behavior, infant feeding, breastfeeding experience, support, weaning, health of the child, and the mother’s feeding habits. The third telephone interview (V5) was performed at the child’s 6th month of life. The fourth telephone interview (V6), with the same questions as V5, contents of breastfeeding habits, infant feeding, weaning, health of the child and nutritional habits of the mother, was realized at the 12th month of the child’s life.

### Ethics

The study was approved by the ethics committee of the Faculty of Medicine of the Philipps University of Marburg and the Justus-Liebig University of Giessen through a positive ethics vote.

### Statistical analysis

Skin contact is assumed to be a predictor in the form of two types of measurement levels. Firstly, it is an ordinal variable with gradations in categories, and secondly, it is a dichotomous variable (yes/no). The influence of skin contact was examined with respect to both target variables. In the case of a metric variable comparing skin contact, the t-test is calculated in the case of a normal distribution and the Mann-Whitney U-test in the case of a non-normal distribution. The groups with and without skin contact are examined regarding dichotomous variables by means of cross-tabulation and tested for significance with Fisher’s exact test. For the dichotomous variables, Fisher’s exact test is calculated, and for the metric variables, the Mann-Whitney U-test or t-test is calculated. Categorical variables with multiple categories are tested for significance using the chi-squared test. The more complex models were applied for the two main predictors, breastfeeding duration and atopic dermatitis.

The following possible confounders related to breastfeeding are analyzed in this association study. The assumed factors influencing the duration of breastfeeding are maternal age, breastfeeding preference, anesthesia type, first postpartum delivery, partner’s attitude toward breastfeeding, hospital breastfeeding instruction, medication, smoking behavior, maternal education level, and midwifery care. The possible influencing factors are examined as control variables using Cox regressions.

Breastfeeding duration is defined as a censored survival time variable, with time to the end of complete breastfeeding as the first main outcome variable. For the descriptive analysis of breastfeeding duration, the Kaplan-Meier survival curve estimator is calculated, and the result is presented as a Kaplan-Meier survival curve. The number of censored cases was about the same in all groups, fulfilling the requirements for this analysis. The log-rank test is used as the significance test. The significance level is set at 5%. In addition, the risk difference between the two groups is estimated using the hazard ratio with a confidence interval of 95%.

There are several factors associated with the 2nd outcome. The present study investigated possible associations between the development of atopic disease and antibiotic administration before birth, the sex of the child, the nutrition of the child, the diet of the mother, the mother’s smoking behavior, the child’s passive smoke exposure, familial atopy burden, and contact with certain animals. These influencing factors were examined as control variables using single logistic regressions. The results of the single logistic regressions are presented using odds ratio, Nagelkerke’s R² and a 95% confidence interval.

## RESULTS

**Figure 2:**
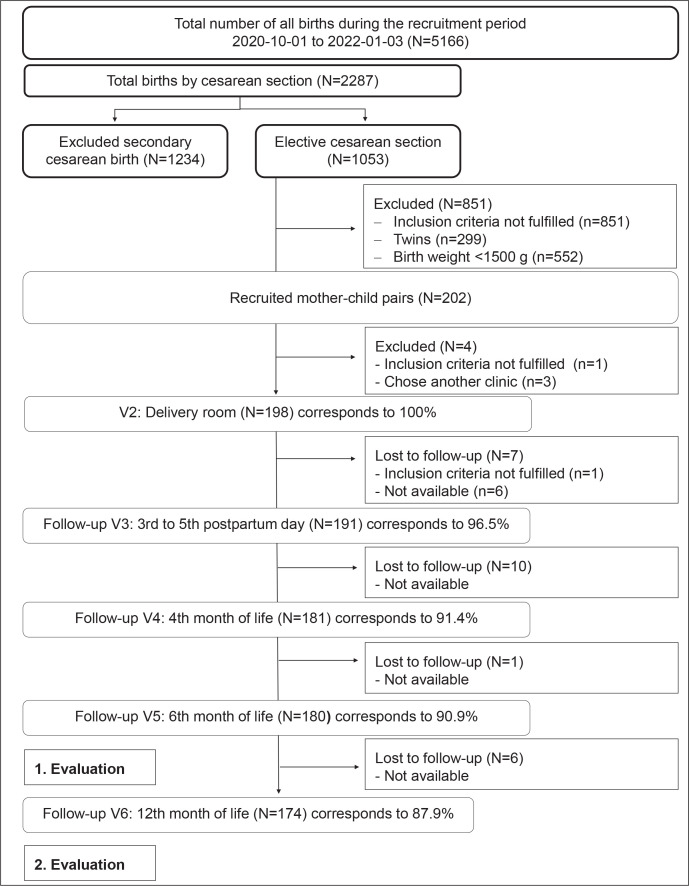
Flowchart shows how the study population developed at the individual measurement times

A total of 202 mother–child pairs were recruited. The data from the checklist were collected from 198 mother–child pairs in the delivery room immediately after birth. The flowchart (Figure 2) shows how the study population developed at the measurement points. At the follow-up at 12 months, the loss rate was 12.12%.

Based on the results, we divided the participants into two groups; 46.6 % of the mother–child pairs had skin-to-skin contact immediately after birth. Table 1 shows the comparison of the two study groups in their respective variables. There is no significant difference between the groups regarding gravida, para, gestational age, and sex of the child.

**Table 1:** Comparison of the groups with and without skin-to-skin contact regarding demographic data, Giessen/Marburg, 2023 (N=198)

*Characteristics*	*Group without skin contact (N=102)^a^*		*Group with skin contact (N=89)^a^*	
	*n*	*%*	*n*	*%*
**Pregnancy**				
2	39	38.2	29	32.6
3	16	15.7	29	32.6
1	22	21.6	14	15.7
4	16	15.7	7	7.9
5	6	5.9	4	4.5
6	3	2.9	4	4.5
7	0	0.0	2	2.2
**Birth**				
2	49	48.0	35	39.3
1	31	30.4	26	29.2
3	19	18.6	24	27.0
4	3	2.9	4	4.5
**Gestational age** (weeks)				
39	51	50.0	60	67.4
38	35	34.3	18	20.2
40	11	10.8	7	7.9
36	0	0.0	2	2.2
37	4	3.9	1	1.1
41	1	1.0	1	1.1
**Gender of the child**				
Male	53	52.0	57	64.0
Female	49	48.0	32	36.0

a 100% corresponds to N=191 mother–child pairs.

In our study, 49.2% of the mothers started full breastfeeding in the hospital. Fifteen mothers did not know if their newborns received supplementary feeding. The other children were fed with formula food. The group of mothers with skin-to-skin contact breastfed earlier, had a higher breastfeeding rate, were more likely to breastfeed fully, and fed formula less often. In comparison, the group of women without skin-to-skin contact, more frequently did not breastfeed at all. If they did breastfeed, it was for a shorter duration. Table 2 shows these results on breastfeeding rate at the respective time of measurement and in the groups with and without skin contact within the first 30 minutes after birth.

**Table 2:** Breastfeeding rate at the respective time of measurement and in the groups with and without skin contact within the first 30 minutes after birth

*Feeding*	*3–5 days after birth (V3) (N=191)^a^*		*4th month of life (V4) (N=181)^b^*		*6th month of life (V5) (N=180)^c^*		*12th month of life (V 6) (N=174)^d^*	
	*n*	*%*	*n*	*%*	*n*	*%*	*n*	*%*
**Breastfeeding rate^e^**	163	85.3	106	58.6	90	50.0	59	33.9
Group with skin contact	78	47.9	51	48.1	47	52.2	29	49.2
Group without skin contact	85	52.1	55	51.9	43	47.8	30	50.8
**Additional food except breast milk**	97	50.8	23	21.7	71	78.9	58	98.3
Group with skin contact	36	37.1	8	34.8	40	56.3	28	48.3
Group without skin contact	61	62.9	15	65.2	31	43.7	30	51.7
**Not nursed/No breast milk**	28	14.7	75	41.4	90	50.0	115	66.1
Group with skin contact	11	39.3	34	45.3	37	41.1	50	13.5
Group without skin contact	17	60.7	41	54.7	53	58.9	65	56.5

a 100% corresponds to N=191 mother–child pairs.

b 100% corresponds to N=181 mother–child pairs.

c 100% corresponds to N=180 mother–child pairs.

d 100% corresponds to N=174 mother–child pairs.

e Breastfeeding rate is the number/percent of mothers who were nursing or feeding their newborn with expressed breast milk at the respective time of measurement.

**Figure 3:**
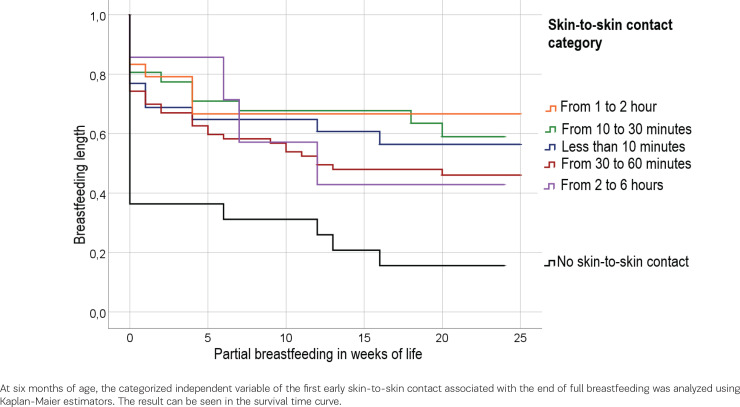
Breastfeeding duration: time at which the mother partially breastfeeds the infant (censored variable) as a function of skin-to-skin contact (categorical variable)

Figure 3 demonstrates that skin-to-skin contact within the first 10 minutes of life in 57.7 % of breastfeeding rates at six months of age. We found if skin-to-skin contact occurred between the first 10 to 30 minutes of life, the breastfeeding rate is 61.3%. The breastfeeding rate in the absence of skin-to-skin contact is 18.2%. The log-rank test for the duration of skin-to-skin contact with breastfeeding duration shows a significant result [χ²(df=4) = 19.617, p<0.001].

The Cox regression of skin-to-skin contact with the breastfeeding rate at the measure point V3 [χ²(df=5) = 18.756, p=0.002] and V5 [χ²(df=5) = 14.143, p=0.015] shows a significant relationship.

Our bivariate analysis with other predictors revealed several significant associations. No significant results were found regarding the age of the mother [χ²(df=189) = 0.063, p=0.950], school education [χ²(df=4) = 2.550, p=0.636], information regarding breastfeeding before pregnancy [HR=1.584; 95% CI: 0.820–3.058, p=0.171], and breastfeeding guidance at the hospital [χ²(df=6) = 3.057, p=0.802].

**Figure 4:**
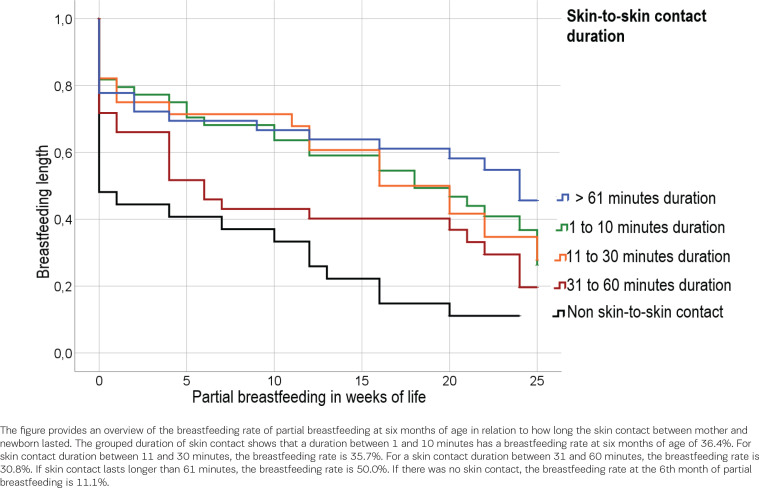
Breastfeeding duration: time when the mother partially breastfeeds the infant, depending on the duration of skin-to-skin contact

We also examined the duration of skin contact in relation to the duration of breastfeeding with the following result: If skin contact lasted longer than 61 minutes, the breastfeeding rate was 50.0%. If there was no skin contact, the breastfeeding rate at six months of partial breastfeeding was 11.1% (95% CI: 3.513–10.043). The log-rank test for the duration of skin contact in minutes with the duration of breastfeeding shows a significant result [χ²(df=4) = 19.617; 95% CI: 12.637–19.740, p<0.001]. Figure 4 shows the duration of breastfeeding at the time when the mother partially breastfeeds the infant, depending on the duration of skin-to-skin contact.

Regarding the 2nd outcome, the question about the occurrence of atopic dermatitis was answered as ‘yes’ by 17 (9.8%) of the mothers, and 7 (43.8%) had a medical diagnosis of atopic dermatitis. One infant had atopic dermatitis by the 4th month of life, and by the 6th month of life, there were nine other infants, all of whom also had atopic dermatitis by the 12th month of life. The time of first onset of atopic dermatitis was at nine months of age in 2 infants, 11 months in 4 infants, and at 12 months in 1 infant. Various other problems were mentioned by the mothers, including itchy skin on the upper arm, dry skin, and multiple conjunctivitis. According to the mothers, as of 12 months of age, there were 26 (15%) children with atopic-allergic conditions. There were ten children with atopic-allergic diseases diagnosed by the physician.

The test for significance of atopic dermatitis within the first 12 months of life with skin-to-skin contact showed no significant association [χ²(df=4) = 1.981, p=0.739, V=0.352). There was no significant association between breastfeeding duration at six months of age and the occurrence of atopic dermatitis at 12 months of age (p>0.05). The comparison between skin-to-skin contact with an infant’s atopic diseases within the first 12 months of life, showed no significant association [χ²(df=10) = 17.971, p=0.055, V=0.227]. The results of the association test for significance using single logistic regressions for each variable between the occurrence of atopic dermatitis within the first 12 months of life with other possible influencing variables, show no significant associations. More detailed results are listed in Tables 3 and 4.

**Table 3:** Significance test for atopic dermatitis in infants in connection with skin contact (dichotomous and categorical)

*Measurement time: 12th month of life (V6) (N=174)^a^*				
*Skin contact: categorical variable*	*χ²*	*Degrees of freedom (df)*	*Significance with χ²-test*	*Cramers-V (V)*
Atopic dermatitis medical diagnosis	1.98	4	0.74	0.35
Atopic dermatitis anamnestic data	9.28	5	0.10	0.23
Asthma	13.98	5	0.02	0.28
Food allergy	5.70	5	0.34	0.18
Summation of atopic diseases in infants at 12 months of age	17.97	10	0.06	0.23
Summation of atopic diseases in infants at 12 months and six months of age	25.62	20	0.18	0.18
Otitis media	13.60	5	0.02	0.28
**Skin contact: dichotomous variable**			**Significance with Fischer‘s exact test**	
Atopic dermatitis medical diagnosis			0.315	
Atopic dermatitis anamnestic data			> 0.999	
Asthma			0.501	
Food allergy			0.093	
Otitis media			0.383	

a 100% corresponds to N=174 mother–child pairs.

**Table 4:** Significance test to compare the development of atopic dermatitis with other possible influencing variables, using single logistic regressions

	*Significance*	*OR*	*95% CI*	*Nagelkerkes R²*
**Measurement time: Immediately after the birth in the delivery room** (N=198)^a^				
Mothers age	0.34	1.07	0.94–1.21	0.01
Sex of the child	0.94	0.95	0.29–3.12	0.001
Preoperative administration of antibiotics	0.06	0.25	0.06–1.05	0.00
**Measurement time: 3–5 days after birth (V3)** (N=191)^b^				
Breastfeeding rate	0.77	0.79	0.16–3.85	0.001
Family history of atopy: predisposition of the mother	0.24	>0.999	-	0.2
Family history of atopy: predisposition of the father	0.46	0.71	-	0.04
Mother smokes prepartum	0.74	1.44	0.17–12.26	0.02
Mother smokes postpartum	1	0.00	-	0.04
Mother‘s medication	0.36	1.78	0.52–0.61	0.01
Mother‘s diet	0.06	3.97	0.96–16.51	0.01
**Measurement time: 4th month of life (V4)** (N=181)^c^				
Breastfeeding rate	0.97	0.98	0.3–3.20	0.00
Other health problems of the child	0.35	1.75	0.54–5.68	0.01
Nutrition of the child	1	0.00	-	0.09
Food other than breast milk	0.71	0.67	0.08–5.49	0.002
Mother smokes	1	0.00	-	0.004
Smoking in the living area	0.726	1.250	0.360–4.35	0.002
Medication of the mother	0.655	0.754	0.218–2.60	0.003
**Measurement time: 6th month of life (V5)** (N=180)^d^				
Breastfeeding rate	0.55	1.43	0.44–4.7	0.005
Nutrition of the child: Food other than breast milk	0.62	0.64	0.12–3.61	0.006
**Measurement time: 12th month of life (V6)** (N=174)^e^				
Breastfeeding rate	0.97	0.97	0.28–3.37	0.000
Nutrition of the child: Food other than breast milk	>0.999	<11.00		0.006

a 100% corresponds to N=198 mother–child pairs.

b 100% corresponds to N=191 mother–child pairs.

c 100% corresponds to N=181 mother–child pairs.

d 100% corresponds to N=180 mother–child pairs.

e 100% corresponds to N=174 mother–child pairs.

## DISCUSSION

The first important result of this study is that most mothers with skin-to-skin contact are more likely to breastfeed exclusively and to breastfeed fully for a longer period. These findings agree with the current study situation and show that skin contact has a positive effect on initial breastfeeding rates^27^. In clinics where skin-to-skin contact was implemented, the initial breastfeeding rate of exclusive breastfeeding was doubled^13,14^. As a result of the prospective cohort study by Guala et al.^14^ on the comparison of skin contact between mother and child in relation to breastfeeding duration, skin-to-skin contact was named as a beneficial factor related to exclusive breastfeeding after cesarean section. It is known that skin contact has a positive effect on exclusively breastfeeding.

The second important result of the present study is that if the first early skin-to-skin contact occurs, the earlier the newborn is first laid on for breastfeeding and the longer the infant is exclusively breastfed. This association is confirmed by other studies. The women who had early skin-to-skin contact with their newborns also breastfed earlier^15,28-30^. It is known that both the first early skin-to-skin contact and the first latch-on have an influence on how long the mother breastfeeds her infant. In summary, mothers who want to breastfeed and who are supported in doing so, by enabling skin-to-skin contact and by early encouragement of breastfeeding, will breastfeed their infants longer.

The third important result of the present study is that skin contact has neither a positive nor a negative effect on the development of atopic-allergic diseases within the first 12 months of life. In the present study, 9.8% of the children developed atopic dermatitis. The fact that fewer children developed atopic dermatitis than described in the literature could be due to regional differences. Another reason could be that the recruitment took place during a period when the COVID-19 pandemic had already lasted nine months. This is an effect that should not be underestimated. To what extent the pandemic overall social situation plays a role as a possible influencing factor on the pathogenesis of atopic-allergic diseases, is unclear. It is possible that less social contact could lead to less sensitization of the immune system^3,31^. It is possible that altered immune responses occur in these contexts that have not yet been explored.

The fourth important result of the present study is that there is no demonstrable association between breastfeeding within the first year of life and the development of atopic-allergic diseases. This may be because in the present study, there was a small number of children with the disease, the breastfeeding rate was lower, or the follow-up was too short, so the evidence could not be provided. Breastfeeding has been found to have a protective effect on the development of allergies in infants at higher risk, and, in contrast, breastfeeding has no protective effect in infants at low risk of allergy^32^. The risk of developing atopic disease increased in children whose mothers breastfed for a shorter period. Children who were not exclusively breastfed had a higher risk of developing atopic dermatitis compared to those who were exclusively breastfed for four months of life^33^.

It is discussed up to which month of life exclusive breastfeeding should be carried out to achieve a protective effect. According to one study, exclusive breastfeeding up to 4 months of age has a protective effect, and breastfeeding up to 7 months of age does not achieve any protective effect beyond that^5^. The current S3 guideline Allergy Prevention (2022) recommends a duration of exclusive breastfeeding for the first 4–6 months of life^34^. The long-term protection of breastfeeding has rarely been studied. It is believed that the protective effect of breastfeeding decreases during childhood and that children who were breastfed for four months, for example, even have a higher risk of developing atopy from the 7th year^35^.

The considerations regarding case number planning proved to be unfeasible in clinical operations about the desired case number level. A study with adequate time and human resources for a recruitment size of well over 1800 participants was not feasible. The results would have been stronger with a higher case number of study participants and/or the formation of a control group with, for example, the birth mode of vaginal delivery. It is possible that the results are not transferable to the total population.

## CONCLUSIONS

This study contributes to the understanding of the relationship between skin-to-skin contact and breastfeeding duration, and breastfeeding duration and the development of atopic-allergic diseases in the 1st year of life. Given the benefits of breastfeeding, skin-to-skin contact should be offered to all mother–infant pairs after birth. Bonding and early breastfeeding are worthy of support. The introduction of a holistic approach to pre-, peri- and post-operative care may be useful.

## Data Availability

The data supporting this research are available from the authors on reasonable request.
